# MethSemble-6mA: an ensemble-based 6mA prediction server and its application on promoter region of LBD gene family in Poaceae

**DOI:** 10.3389/fpls.2023.1256186

**Published:** 2023-10-09

**Authors:** Dipro Sinha, Tanwy Dasmandal, Krishnayan Paul, Md Yeasin, Sougata Bhattacharjee, Sneha Murmu, Dwijesh Chandra Mishra, Soumen Pal, Anil Rai, Sunil Archak

**Affiliations:** ^1^ ICAR-Indian Agricultural Statistics Research Institute, Delhi, India; ^2^ Graduate School, ICAR-Indian Agricultural Research Institute, Delhi, India; ^3^ ICAR-National Bureau of Fish Genetic Resources, Lucknow, India; ^4^ ICAR-National Institute for Plant Biotechnology, Delhi, India; ^5^ ICAR-Indian Agricultural Research Institute, Hazaribagh, Jharkhand, India; ^6^ Indian Council of Agricultural Research, Delhi, India; ^7^ ICAR-National Bureau of Plant Genetic Resources, Delhi, India

**Keywords:** 6mA, ensemble model, DNA methylation, MethSemble-6mA, LBD gene, wheat, poaceae, prediction

## Abstract

The Lateral Organ Boundaries Domain (LBD) containing genes are a set of plant-specific transcription factors and are crucial for controlling both organ development and defense mechanisms as well as anthocyanin synthesis and nitrogen metabolism. It is imperative to understand how methylation regulates gene expression, through predicting methylation sites of their promoters particularly in major crop species. In this study, we developed a user-friendly prediction server for accurate prediction of 6mA sites by incorporating a robust feature set, viz., Binary Encoding of Mono-nucleotide DNA. Our model,MethSemble-6mA, outperformed other state-of-the-art tools in terms of accuracy (93.12%). Furthermore, we investigated the pattern of probable 6mA sites at the upstream promoter regions of the LBD-containing genes in *Triticum aestivum* and its allied species using the developed tool. On average, each selected species had four 6mA sites, and it was found that with speciation and due course of evolution in wheat, the frequency of methylation have reduced, and a few sites remain conserved. This obviously cues gene birth and gene expression alteration through methylation over time in a species and reflects functional conservation throughout evolution. Since DNA methylation is a vital event in almost all plant developmental processes (e.g., genomic imprinting and gametogenesis) along with other life processes, our findings on epigenetic regulation of LBD-containing genes have dynamic implications in basic and applied research. Additionally, MethSemble-6mA (http://cabgrid.res.in:5799/) will serve as a useful resource for a plant breeders who are interested to pursue epigenetic-based crop improvement research.

## 1 Introduction

Plant architecture is an important trait that distinguishes domesticated plant types from wild ones and enables breeders to choose the most productive types for agriculture. One of the major gene families that influence plant architecture is the family of Lateral Organ Boundaries Domain (LBD) genes. In plant systems, LBD genes have a wide role from embryonic development to stress resistance (Zhao et al., 2023). The role of LBD-containing genes has been reported in various agricultural crops including ideotype in rice (Zhao et al., 2023); drought tolerance in maize (Jiao et al., 2022), tomato (Liu et al., 2020), and potato (Liu et al., 2019); salt tolerance in switch grass (Guan et al., 2023); and multiple abiotic stress tolerance in wheat (Wang et al., 2021) and cotton (Yu et al., 2020).

In addition to studying the structure and function of genes, it is essential to unravel gene regulation to achieve expected plant architecture and better quantity and quality of produce. Genes in eukaryotes are regulated, among others, by epigenetic factors including stress memory. Conrad Waddington introduced the field of epigenetics in 1942, which explores heritable and reversible alterations in gene expression without modifications to the DNA sequence (Waddington, 2012). The involvement of these modifications in plants’ gene regulatory mechanism has been revealed in recent studies for both biotic (Ashapkin et al., 2020) and abiotic stresses (Saraswat et al., 2017). Epigenetic mechanisms encompass diverse biological processes such as DNA methylation, histone modification, and chromosome remodeling, among which DNA methylation is considered a fundamental and widely distributed epigenetic process in various animal genomes, directly impacting gene expression (Ratel et al., 2006). DNA methylation can be categorized based on the location of methylation as *N* ^6^-methyladenine (6mA) (O’Brown and Greer, 2016), *N* ^4^-methylcytosine (4mC), and 5-methylcytosine (5mC) (Zhou et al., 2018; Lv et al., 2019; Lv et al., 2020). Although many studies have been performed on methylated cytosine, the potency of 6mA methylation is yet to be unveiled thoroughly. As per the literature, 6mA plays a vital role in basic cell functions such as replication (Campbell and Kleckner, 1990), transcription (Robbins-Manke et al., 2005), and repair (Pukkila et al., 1983). Although its presence can be observed in all three kingdoms of life, the distribution pattern of 6mA sites throughout the genome does not occur randomly, making it essential to accurately identify the specific locations of 6mA positions across the entire genome.

In recent years, there has been significant progress in high-throughput sequencing techniques, enabling the study of DNA 6mA modifications on a genome-wide scale. For instance, a method combining bisulfite sequencing with methyl-DNA immunoprecipitation was developed to identify 6mA sites in eukaryotes (Pomraning et al., 2009). Another efficient technique involved capillary electrophoresis and laser-induced fluorescence to quantify global adenine methylation of DNA (Krais et al., 2010). Additionally, the single-molecule real-time (SMRT) sequencing technology was utilized to detect genome-wide positions of 4mC and 6mA throughout the entire genome (Flusberg et al., 2010). Mass spectrometry analysis and 6mA immunoprecipitation followed by sequencing (IP-seq) were also employed to decipher 6mA sites in the rice genome (Zhou et al., 2018). However, these approaches have three significant limitations: time-consuming, labor-intensive, and expensive.

For bypassing these issues, *in silico* prediction tools can provide a faster and more reliable alternative to these *in vitro* methods. The ground-breaking research originated in 2019 when Chen et al. introduced a classifier called i6mA-Pred, based on a support vector machine (SVM), which was developed using a feature space consisting of nucleotide frequencies and nucleotide chemical properties. This classifier was trained and evaluated on a benchmark rice dataset comprising 880 6mA sites and 880 non-6mA sites obtained from the rice genome (Chen et al., 2019). So far, a few other attempts have been made to predict these modifications in plants, viz., iDNA6mA (Tahir et al., 2019), SDM6A (Basith et al., 2019), iDNA6mA-Rice (Lv et al., 2019), SNNRice6mA (Yu and Dai, 2019), i6mA-DNCP (Kong and Zhang, 2019), and i6mA-Caps (Rehman et al., 2022) for rice (Huang et al., 2020) and *Arabidopsis* (Wang and Yan, 2018). A major shortcoming of these tools is that they are based on single species, and cross-species performance is not well known. Only Meta-i6mA (Hasan et al., 2021), proposed in 2021, has provided a significant result in cross-species.

Keeping these lacunas in mind, in our first attempt, we developed an ensemble-based model called EpiSemble (Sinha et al., 2023) based on two model plant species, viz., *Oryza* and *Arabidopsis*. It outperformed the state-of-the-art tools for all the evaluation measures. In the present study, we improved our model by adding more robust features set, viz., binary encoded nucleotide frequencies, and we could achieve better performance in terms of accuracy while applying it in cross-species analysis. In order to study the regulation of complex genes and gene families, we chose LBD genes, as they have a crucial role in both developmental and stress conditions and are conserved across species (Wang et al., 2021; Xu et al., 2021). We investigated the distribution pattern of the 6mA sites in the promoter region, in the four species of *Triticum*, one species of *Oryza*, and one species of *Arabidopsis*. Here we report the mapping the 6mA sites of selected LBD domain-containing genes to understand their functionality and conservation across species.

## 2 Materials and methods

Section 2.1 deals with the materials and methods used for the construction of the prediction model, and Section 2.2 deals with the materials and methods employed for the analysis of methylation of promoter regions of LBD genes.

### 2.1 Construction of prediction model

The prediction model consisted of three modules: vectorization of DNA fragments, feature set optimization using a hybrid feature selection module, and finally, ensemble modeling. The aim was to include as many features as possible in the model to obtain higher prediction accuracy.

#### 2.1.1 Dataset description

All the datasets was downloaded from public domains. For the training of the machine learning models, we selected benchmark datasets of RiceLv (sub sp) and *Arabidopsis* thaliana (Hasan et al., 2021) and the test dataset was of rice Nipponbare (http://www.elabcaas.cn/smep/index.html). The details of positive and negative 6mA samples in these datasets are given in **Table 1**. Size of all the positive and negative DNA fragments was 41 bp (Wang et al., 2021). The total rice and *Arabidopsis* dataset was used for training purposes, and the Nipponbare dataset was used for testing purposes. This approach provides a validation of the application of the developed model in intra-specific species.

**Table 1:** Description of the datasets used for model construction and validation.

Data Class	Training data		Testing data (Nipponbare)
	RiceLv	*Arabidopsis*	
**Positive**	154,000	31,873	5,000
**Negative**	154,000	31,873	10,000
**Total**	308,000	63,746	15,000

#### 2.1.2 Feature extraction of DNA sequences

DNA sequences need to be vectorized before the machine learning module can be applied. For this, five feature vectors were considered. Dinucleotide frequency (DNF) has been proven to be an efficient feature for converting short DNA fragments (Hasan et al., 2021). It also helps to reduce the time complexity of the computation, as it comprises less vector space, i.e., 16 in comparison with higher-order nucleotide frequencies (tri-, tetra-, hexa-, etc.). Another feature is nucleotide chemical properties (NCPs), where the bases give a score based on their physio-chemical properties like ring structures (single or double), hydrogen bonds (two or three), and base composition (amino or keto). Based on this, the four bases are represented as (1, 1, 1), (0, 0, 1), (1, 0, 0), and (0, 1, 0) for adenine, cytosine, guanine, and thymine, respectively (Chen et al., 2019). It can be also noticed that guanine–cytosine (GC) content varies in different DNA fragments based on their roles. In this study, a log-transformed GC content was used. Transformation is performed to reduce the weight of the GC content feature compared to other feature sets (Sinha et al., 2022). Another recently used feature is the Average Mutual Information Profile (AMIP), where the AMI measures the level of “information” that can be obtained from the relationship between two random variables, X and Y (X and Y are the two DNA sequences here) (Bauer et al., 2008). In the context of genomic sequences, X and Y represent nucleotide bases. Therefore, the proposed genomic signature is a vector where each entry corresponds to the AMI between nucleotides that are a certain number of positions apart. The AMI profile provides a summary of the statistical dependencies between nucleotides at different distances within the sequence (Eq. 1). To put it simply, the AMI profile is a way to represent the amount of shared information between nucleotides separated by specific distances in a genomic sequence.


(1) $$MI_{k}=\sum_{X\in S}\sum_{Y\in S}p_{k}\left(X,Y\right)log\frac{p_{k}\left(X,Y\right)}{p\left(X\right)p\left(Y\right)}.$$

Here, *p_k_* is the probability of two nucleotides occurring together at *k* distance apart.

Along with these features, another robust feature for encoding DNA sequences, Binary Encoding of Mono-nucleotide DNA (MBED), was incorporated into the model. MBED was proven to be an efficient representer in the case of cross-species. In this, the four nucleotides A, C, G, and T are represented as (1000), (0010), (0100), and (0001) respectively (Wang et al., 2021).

#### 2.1.3 Selection of informative features

The feature set contains both relevant and irrelevant features. Irrelevant features may lead to improper training of the models. In previous studies, feature selection techniques like Maximum Relevance Maximum Distance (MRMD) (Chen et al., 2019) and Sequential Forward Selection (SFS) (Basith et al., 2019) were implemented. To obtain more robust features, a hybrid feature selection module was used in this study combining random forest and stepwise regression (Chen and Howard, 2015).

#### 2.1.4 Machine learning models

Based on the performance of these two datasets, three machine learning models were chosen, viz., SVM (Cortes et al., 1995), random forest (RF) (Breiman, 2001), and gradient boosting (GB) (Friedman, 2001). SVM tends to find the optimum hyperplane between 6mA and non-6mA sequences. In this study, radial function [ $k\;\left(x_{i},x_{j}\right)={\left(-\gamma |x_{i}-x_{j}\right)}^{2}$ ] was taken as a kernel with the “C-classification” type. R-package “e1071” was used to implement SVM. For RF, the number of trees was set to 500 with five splits. R-package “randomForest” was used for RF. In the case of GB, the stochastic method was chosen with an interaction depth of 3 and shrinkage value of 0.1 and 150 ntrees. It was implemented using the “gbm” R-package.

#### 2.1.5 Ensemble model

**Figure 1:**
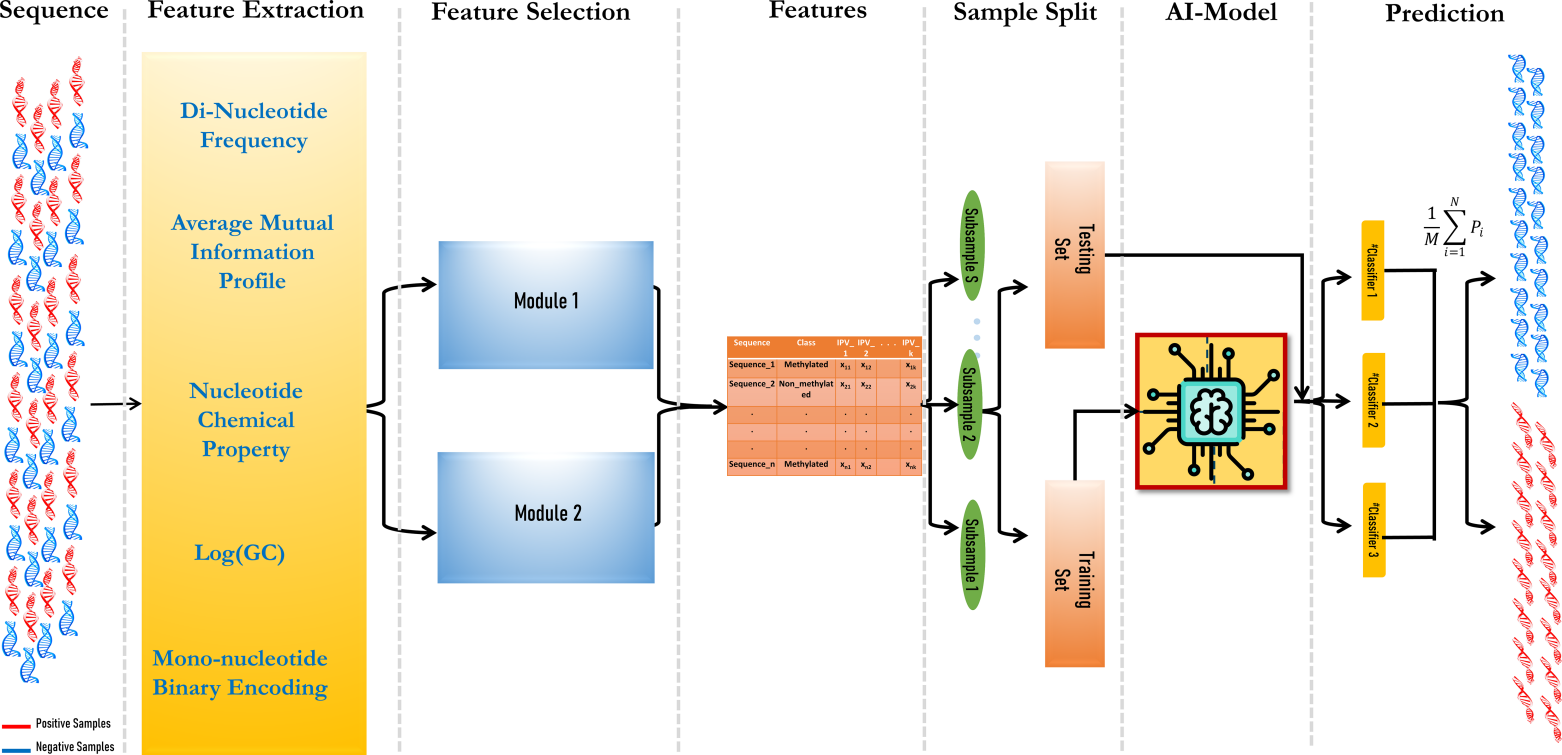
Workflow of the MethSemble-6mA model.

To integrate the prediction from each model, ensemble voting was used. Here, an instance with the highest vote, which means with at least two predictions favoring it, was chosen. The final prediction was computed as follows using the prediction score (Eq. 2):


(2) $$Prediction=\frac{1}{M}\sum_{i=1}^{M}P_{i},$$

where *M* is the number of ML models, and *P_i_* is the prediction value, i.e., whether it is methylated or not of each ML model. The workflow of the model is given in **Figure 1**.

#### 2.1.6 Performance evaluation

As in the other experiments, evaluation metrics for the classifiers included sensitivity, specificity, accuracy, and Matthews’ correlation coefficient (MCC) (Basith et al., 2019; Lv et al., 2019; Yu and Dai, 2019; Huang et al., 2020; Kha et al., 2022; Rehman et al., 2022; Sinha et al., 2023).

The proportion of positively tagged cases that are projected to be positive is termed as sensitivity.


$$\text{Sensitivity}=\frac{P_{+}^{+}}{P_{+}^{+}+P_{+}^{-}}$$

The proportion of negatively tagged cases that are projected to be negative is termed as specificity.


$$\text{Specificity}=\frac{P_{-}^{-}}{P_{-}^{-}+P_{-}^{+}}$$

The ratio of successfully identified cases to all test data points is known as accuracy.


$$\text{Accuracy}=\frac{P_{+}^{+}+P_{-}^{-}}{P_{+}^{+}+P_{-}^{-}+P_{-}^{+}+P_{+}^{-}}$$

Between the actual and predicted series, there is a correlation known as the MCC. It returns numbers between −1 and +1. A value of 0 is similar to a random forecast, while a coefficient of −1 signifies a full difference between the prediction and the observation. A coefficient of +1 denotes a flawless prediction. The MCC can be calculated directly from the confusion matrix by the following formula:


$$\text{MCC}=\frac{P_{+}^{+}\times P_{-}^{-}-P_{-}^{+}\times P_{+}^{-}}{\sqrt{\left(P_{+}^{+}+P_{-}^{+}\right)\left(P_{+}^{+}+P_{+}^{-}\right)\left(P_{-}^{-}+P_{-}^{+}\right)\left(P_{-}^{-}+P_{+}^{-}\right)}}$$

where

$P_{+}^{+}$ = Instances that are true and predicted as true.

$P_{-}^{-}$ = Instances that are false and predicted as false.

$P_{-}^{+}$ = Instances that are false but predicted as true.

$P_{+}^{-}$ = Instances that are true but predicted as false.

The receiver operating characteristic (ROC) curve was also used to evaluate the effectiveness of this strategy. One of the most important measures of a binary classifier’s effectiveness is the area under the ROC curve (AUC), which is determined by graphing the true positive rate (sensitivity) against the false-positive rate (1 − specificity). Better predictions are produced when the value is closer to 1, whereas a value of 0.5 indicates random prediction.

### 2.2 Promoter analysis of LBD genes

Upstream promoter regions (1.5 kb) of LBD-containing genes of six crop species including monocot species (*T. aestivum* (Wang et al., 2021), *Triticum dicoccoides*, *Triticum urartu*, *Aegilops tauschii*, and *Oryza sativa*) and dicot species (*Arabidopsis thaliana* (Xu et al., 2021)) were selected for this study (**Table 2** **)**. The basis of selection was to capture representative species (*O. sativa* acted as a reference for the Poaceae family, while *A. thaliana* acted as a non-grass comparison). These sequences are available with high sequencing coverage, which will provide a more informative prediction of our analysis. In order to understand the dynamics of 6mA sites through evolution and speciation, our findings on wheat were emphasized, and therefore, four out of six species were selected from the Triticeae family.

**Table 2:** Species-wise frequency of identified LBD genes.

Species	Number of LBD genes
*Triticum aestivum*	94
*Triticum dicoccoides*	49
*Triticum urartu*	27
*Aegilops tauschii*	29
*Oryza sativa*	37
*Arabidopsis thaliana*	43

#### 2.2.1 Extraction of promoter region

Promoter regions of these genes were extracted from the Ensembl Plants database using the Biomart tool (http://plants.ensembl.org/info/data/biomart/index.html). Promoter regions were selected from their respective databases like *Triticum aestivum* genes (IWGSR) for *T. aestivum*, *Triticum dicoccoides* genes (WEWSEQv.1.0) for *T. dicoccoides*, *Triticum urartu* genes (Tu2.0) for *T. urartu*, *Aegilops tauschii* genes (Aetv4.0) for *A. tauschii*, *Oryza sativa indica* group genes (ASM465v1) for *O. sativa*, and *Arabidopsis thaliana* genes for *A. thaliana*. The 1.5-kb upstream region of each gene from the transcription start site (TSS) was extracted.

#### 2.2.2 Pre-processing of promoter sequences

The ambiguous bases (containing “N”) were trimmed out from promoter sequences using the seqkit tool (https://bioinf.shenwei.me/seqkit/). Then, the promoters from all six species were fragmented into 41-bp sequences using the “split fasta” function of sequence Manipulation Suite (https://www.bioinformatics.org/sms2/split_fasta.html) separately. The resulting fasta file was fed into the MethSemble-6mA server for the prediction of the sequences containing 6mA sites.

#### 2.2.3 Prediction of 6mA sites in the promoter of LBD genes

For the prediction of the 6mA sites in the promoter region of the LBD genes, a bidirectional approach was taken into consideration. The prediction was performed by taking both rice and *Arabidopsis* as model plants followed by taking the intersection (Eq. 3) of the two predictions. As in the dataset, both types of data are present, viz., Poaceae and Brassicaceae; these two were used concurrently, which means prediction has been performed by taking rice and *Arabidopsis* as model plants one by one. This will provide a robust prediction and reduce the chance of obtaining false-positive sites.


(3) $$6mA\;\left(LBD\right)=Model_{Rice}\;\cap^{\;}Model_{Arabidopsis}$$

#### 2.2.4 Mapping of 6mA sites and phylogenetic analysis

Predicted 6mA sites found in upstream promoter elements of the LBD-containing genes were filtered for the highest number of sites present and were mapped using the MapChart tool. Standalone BLASTn search was performed using NCBI BLAST+ tool using *T. aestivum* LBD-containing genes under study as query and *T. dicoccoides*, *T. urartu*, and *A. tauschii* LBD-containing genes as database. The obtained hits were filtered with 100% identity for multiple sequence alignment (ClustalW) following phylogenetic analysis (MLM) using MEGAXI (Tamura et al., 2021). The generated Newick file was used for visualizing the tree using the online iTOL tool (https://itol.embl.de/).

#### 2.2.5 Pipeline of 6mA site prediction of LBD-containing genes

**Figure 2:**
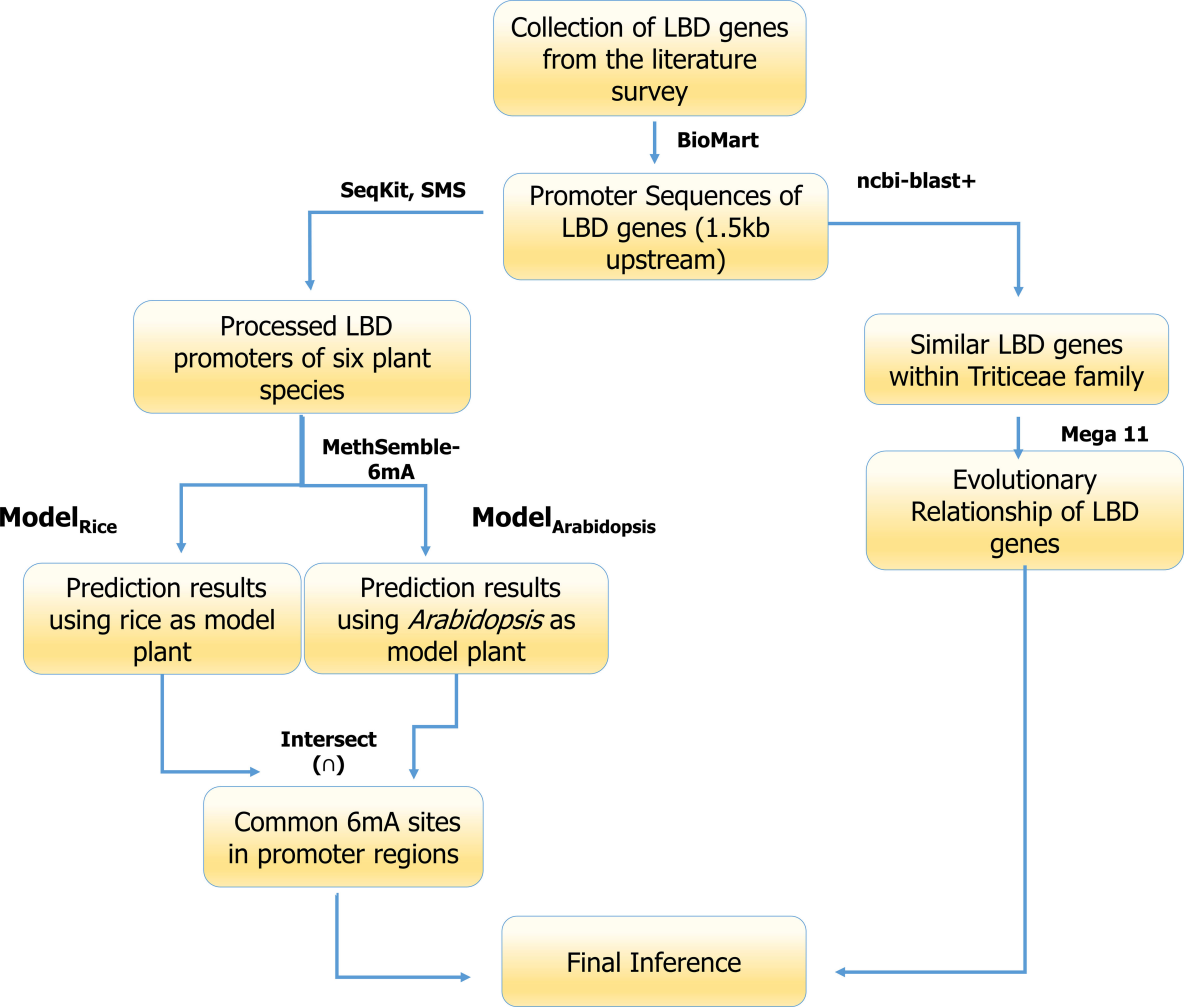
6mA methylation site prediction pipeline used in the current study.

The pictorial representation pipeline used for analysis of the promoters of LBD genes is given as **Figure 2**.

## 3 Results

### 3.1 Prediction of 6mA sites

The prediction was performed based on both rice and *Arabidopsis*. The results are as follows.

#### 3.1.1 Feature space analysis

The feature sets DNF, NCP, AMIP, log-transformed GC content, and MBED resulted in a total vector space of 124 dimensions. The top 40 features were taken from each feature selection module (SwR and RF) (Chen and Howard, 2015), and common features predicted in each module were taken for model development. We found that the final feature space consisted of three features from DNF, eight from NCP, seven from AMIP, one from log-transformed GC content, and nine from MBED, which makes a total feature space of 28 dimensions. Clearly, it can be seen that MBED has the highest contribution in terms of constructing the final feature space, followed by NCP.

#### 3.1.2 Prediction of 6mA sites

**Figure 3:**
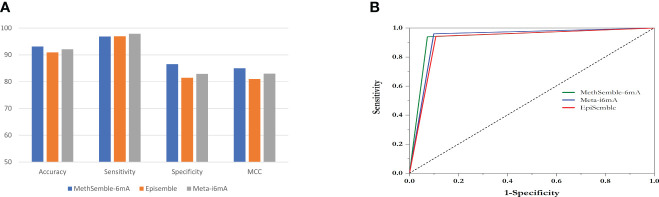
Performance of MethSemble-6mA with existing models. **(A)** Bar Plot of Performance Metrics **(B)** ROC Curve.

Testing was performed using the Nipponbare dataset. It was found that the RF performed better, in terms of accuracy, sensitivity, specificity, MCC, and AUC, than SVM and GB in both cases, i.e., when trained with the rice dataset (**Supplementary Table 1**) and when trained with the *Arabidopsis* dataset (**Supplementary Table 2**). The ensemble model also performed better than the existing models in terms of accuracy, specificity, and MCC, while Meta-i6mA exhibited more sensitivity (**Figure 3A**; **Supplementary Table 3**). Also, in terms of AUC, MethSemble-6mA outperformed the other two state-of-the-art tools (**Figure 3B**). To ascertain the superiority of the MethSemble-6mA model, TOPSIS analysis was performed.

#### 3.1.3 TOPSIS analysis

The performances of multiple tools, assessed on various evaluation criteria, were tested using the TOPSIS method, which aids in multiple-criteria decision-making (MCDM) (Barretta et al., 2023). The R-package “topsis” was employed for this analysis. This approach enabled the determination of the best-ranked tools based on their similarity to the ideal solution across the multiple criteria considered. MethSemble-6mA secured the top rank followed by Meta-i6mA and EpiSemble (**Table 3**). The TOPSIS analysis was performed based on the evaluation measures.

**Table 3:** Ranking of the models using the TOPSIS method.

	Score	Rank
**MethSemble-6mA**	**0.67**	**1**
Meta-i6mA	0.59	2
EpiSemble	0.20	3

Output of our tool showing comparative superior performance highlighted as bold.

#### 3.1.4 MethSemble-6mA server

**Figure 4:**
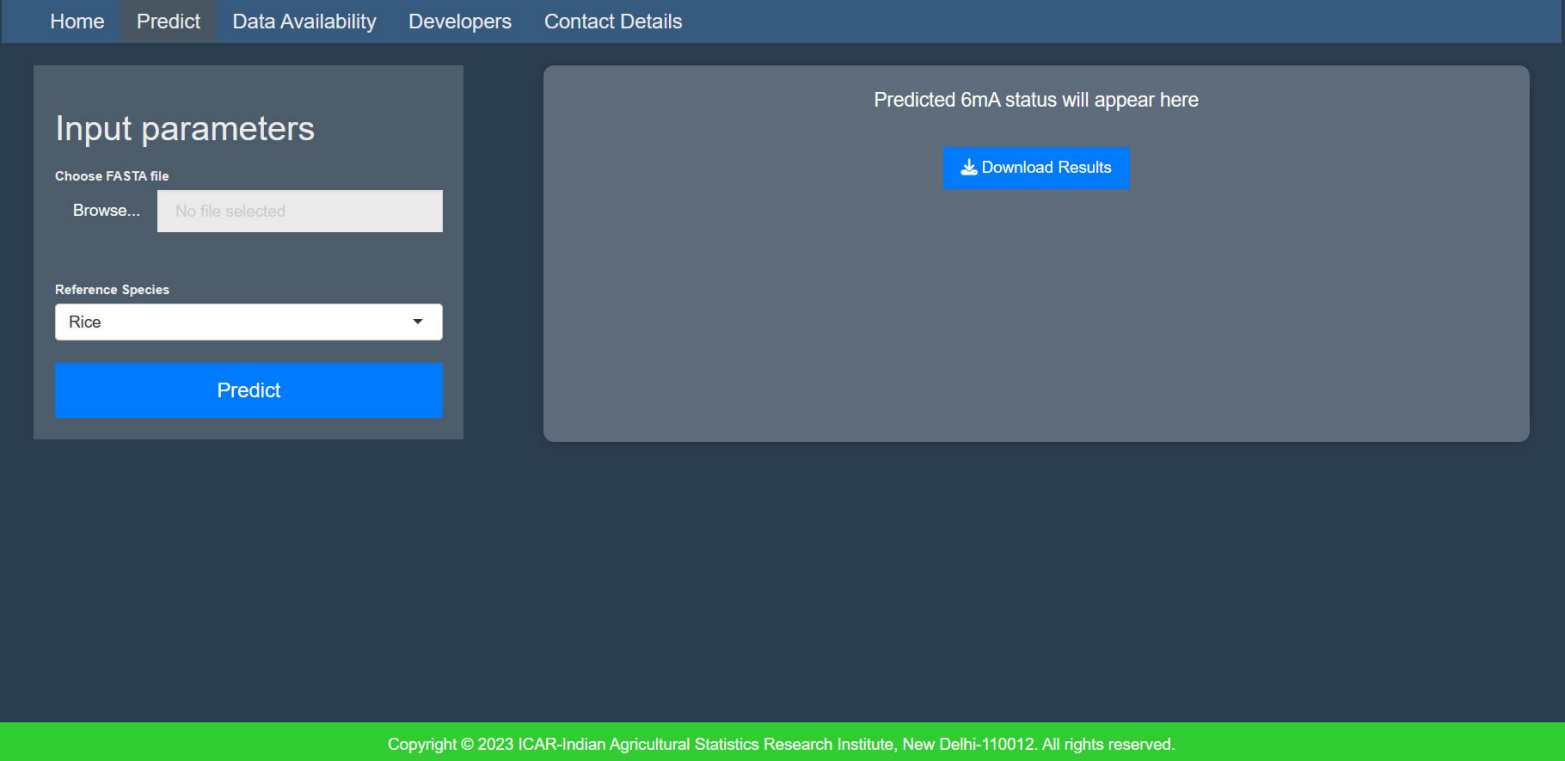
Homepage of MethSemble-6mA.

A user-friendly server was built for hassle-free implementation of the model. The interface is given in **Figure 4**.

- File format: Provide the input file in fasta format.
- Sequence details: The sequence length of the input must be exactly 41nt, and it should not contain any ambiguous bases (“N”).
- Number of inputs: Users can provide multiple sequences in a multifasta file.
- Accession name: The accession name must be different for each sequence in the multifasta file.

### 3.2 6mA analysis of promoter region of LBD genes

#### 3.2.1 6mA prediction in LBD gene promoters

After the fragmentation and removal of ambiguous sequences, a total of 9,614 fragments were obtained from the selected promoters of 279 LBD genes. When prediction was performed by taking rice as a model plant, 1,246 fragments out of 9,614 fragments were found to be methylated. However, when prediction was performed by taking *Arabidopsis* as a model plant, 1,173 fragments out of 9,614 fragments were found to be methylated. After taking the common methylation sites, a total number of 1026 methylated fragments were obtained. Species-wise frequency of 6mA sites is given in **Table 4**.

**Table 4:** Frequency of 6mA sites in upstream promoter regions of LBD-containing genes in selected species under study.

Species	Frequency of 6mA sites
*Triticum aestivum*	322
*Triticum dicoccoides*	188
*Triticum urartu*	145
*Aegilops tauschii*	134
*Oryza sativa*	126
*Arabidopsis thaliana*	134

#### 3.2.2 Distribution pattern of the 6mA sites in LBD gene promoters

**Figure 5:**
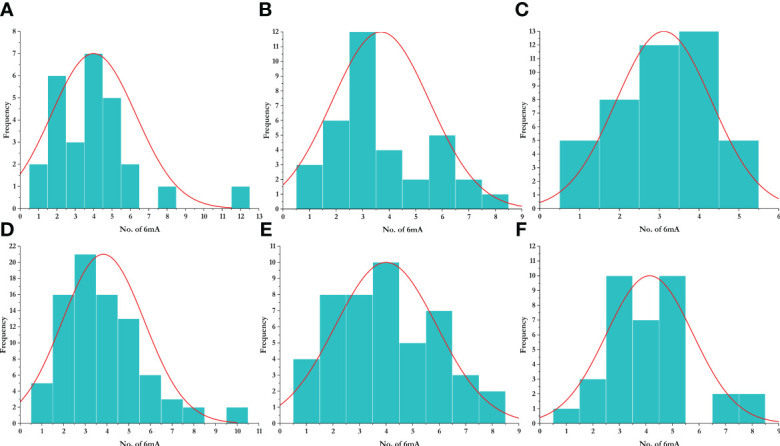
Distribution of 6mA sites in different species. **(A)** *Aegilops tauschii*, **(B)** *Arabidopsis thaliana*, **(C)** *Oryza sativa*, **(D)** *Triticum aestivum*, **(E)** *Triticum dicoccoides*, and **(F)** *Triticum urartu*.

To study the methylation pattern and number of frequencies of 6mA sites, we plotted a histogram, which represents the frequency of the number of promoters that contain a certain number of 6mA sites. We found that most promoters contain three 6mA sites, while the highest number of 6mA sites was found in the promoter region of *Aegilops tauschii* (AET1Gv20706400). The species-wise distribution is given in **Figure 5**. The enrichment analysis of the LBD genes having 6mA sites yielded position-specific nucleotide abundance (**Supplementary Figure 1**).

#### 3.2.3 Map of 6mA sites predicted in the promoter regions of selected LBD-containing genes

6mA sites at upstream promoter regions of the LBD-containing genes under study with the highest occurring frequency were plotted for visualization. The highest frequency obtained was as follows: *O. sativa* (BGIOSGA001373; 8), *A. thaliana* (AT4G37540, AT5G66870, AT4G22700, AT3G27650, and AT2G30340; 5), *T. aestivum* (TraesCS5A02G284000 and TraesCS3D02G340000; 10), *T. urartu* (TuG1812S0002083700.01, TuG1812G0100002612.01, and TuG1812G0500005367.01; 5), *T. dicoccoides* (TRIDC2BG028050 and TRIDC6BG069150; 8), and *A. tauschii* (AET1Gv20706400; 12).

#### 3.2.4 Phylogenetic analysis of LBD-containing genes of *Triticum* species under study

**Figure 6:**
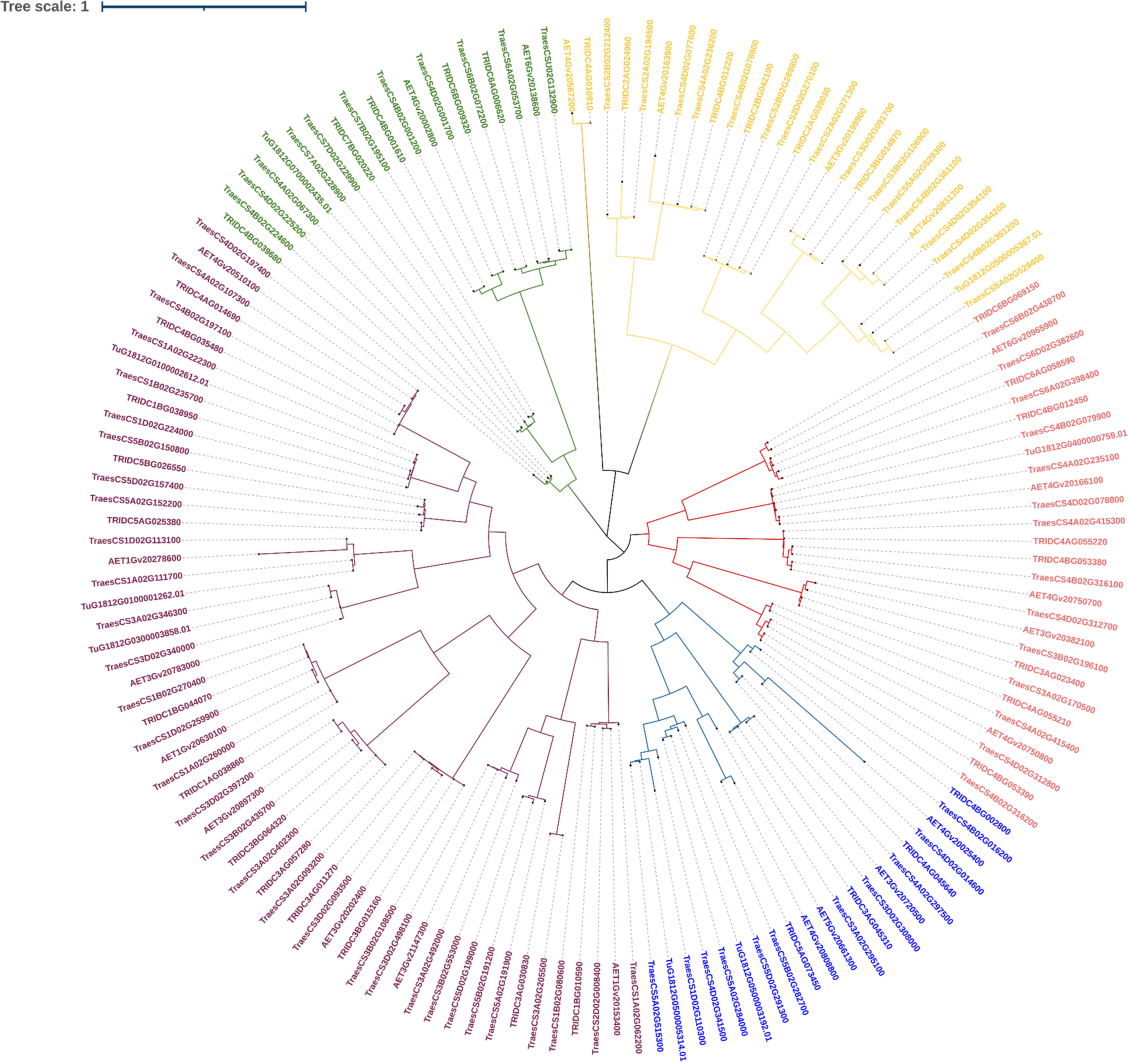
Phylogeny of LBD genes of *Triticum* species.

We shortlisted 100% identity genes with LBD domain identified using BLASTn search. A phylogenetic tree was generated to interpret the evolutionary conservation of 6mA methylation pattern over the course of evolution and speciation in *Triticum* (**Figure 6**). We classified the tree with five clades and analysed for 6mA site conservation. *T. urartu* had the least commonality with other wheat species under study (**Figure 6**). We took a few clade genes for further analysis of the 6mA position and details are given in the discussion section.

## 4 Discussion

LBD genes play a crucial role in plant developmental biology. To our knowledge, there are a few tools available to date that work on multiple species. Our proposed model, MethSemble-6mA, was trained based on both rice and *Arabidopsis* datasets. This model was found to be efficient in testing cross-species based on evaluation measures like accuracy and specificity (**Supplementary Tables 1, 2**). Adding a robust feature like MBED helps to identify the 6mA sites in cross-species. In the case of *Arabidopsis*, the sensitivity is relatively low (**Supplementary Table 2**), which reflects that, although it can detect true-positive (TP) instances, improvement can be performed to decrease false-positive (FP) instances. In our opinion, TP poses a particular pattern in methylation states, as it is not random, and true negative (TN) instances lack that. Further investigation can be performed to reduce the FP instances to make the model more robust. However, MethSemble-6mA is still outperforming other existing cross-species tools in terms of accuracy, specificity, and MCC. TOPSIS analysis of the models based on the evolutionary measures also depicts the same outcome.

Employing Methsemble-6mA, we predicted the 6mA sites based on both the model plant species and took the common sites having the 6mA sites. This will reduce the FP instances but will provide a more precise outcome about the probable 6mA sites. We found that the methylation rate per gene in the Triticeae family (greater than 3.83) is higher than in *Oryza sativa i.e.,* 3.68 and in *Arabidopsis thaliana i.e.,* 3.11. We also observed that, with evolution, the rate of methylation is decreasing within Triticeae family; as we can see the rate of methylation in *Triticum urartu i.e.,* 4.14, and *Aegilops tauschii i.e.,* 4.0 (wild type wheat) is higher than the *Triticum dicoccoides* (cultivated wheat ancestors) *i.e.,* 3.91 and *Triticum aestivum i.e.,* 3.83 (cultivated wheat). It will be interesting to further analyse correlation between ploidy and methylation rate.

**Figure 7:**
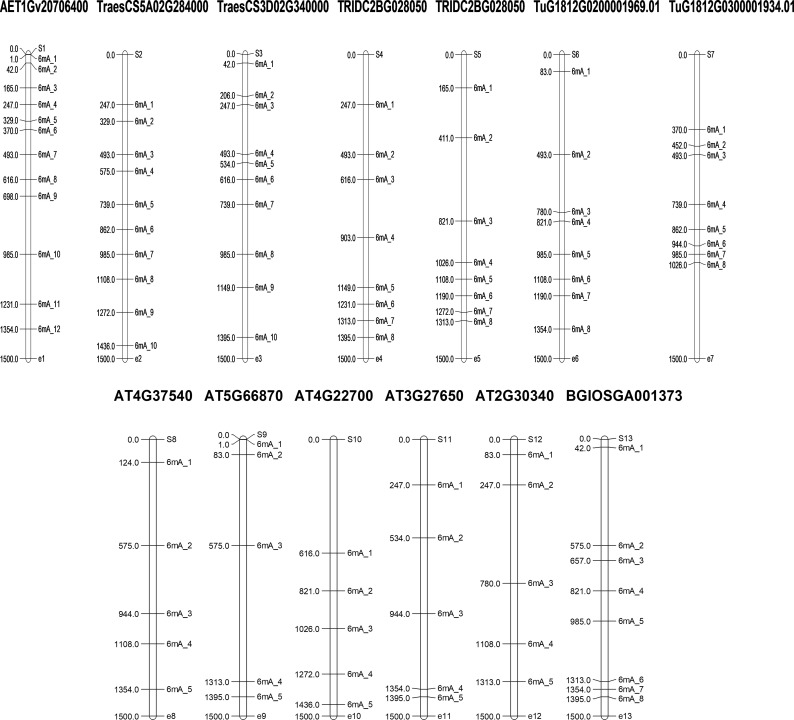
Map of 6mA sites predicted in the promoter regions of selected LBD-containing genes.

In addition to the species-specific differences, we also observed that the methylation rate is much higher in the 700–1,300-bp upstream (from TSS) compared to other parts of the promoter (**Figure 7**; **Supplementary Material 1**) except for AET1Gv20706400, where initial 700 bases are rich in 6mA sites and TraesCS5A02G284000, where 6mA sites are evenly distributed throughout the promoter region.

In order to validate our findings, we selected closely related LBD gene pairs under study were selected. These were obtained through all against all blast hits with 100% identity and their predicted 6mA sites in promoter regions were analyzed critically. It was observed that in wheat, with polyploidization, speciation and domestication, 6mA methylation sites have reduced. Moreover, similarity in a few methylation sites also indicates their conservation across species. For example, two phylogenetically closed genes, TRIDC6BG069150 (from *T. dicoccoides*) and TraesCS6B02G438700 (from *T. aestivum*), were analyzed, and it was found that the promoter of the former has eight 6mA sites, the promoter of the latter one has five sites, and two sites, 1026-1066 and 1313-1353, were conserved for both the genes. Again, from the same clade, AET6Gv20965900 (from *A. tauschii*) promoter has six 6mA sites of which 1026-1066 site is conserved in all these three species. Similar inferences can be made using other genes from another clade. For example, promoters of TraesCS3B02G108500, TRIDC3BG015160, and AET3Gv20202400 genes have four, four, and one 6mA sites, respectively, while the first and last two genes have a conserved site at base position at 165-205 and 1313-1353, respectively. This again indicates the conservation of 6mA sites across the domestication of wheat, and it can be an important finding to understand speciation, domestication and gene evolution over time. Moreover, it help understand how gene regulation has changed through changing methylation sites through speciation. Ultimate objective is to identify screening strategy to select climate ready genotypes.

## 5 Conclusion

Targeting the methylation sites in gene regulatory elements to investigate gene expression patterns and genome imprinting mechanisms is always intriguing to obtain more robust information about functional epigenetic sites in the genome. An improved model for predicting 6mA sites, more specifically for Poaceae and Brassicaceae family crops, has been delivered to be utilized in molecular biology research. Nevertheless, a dedicated user-friendly server was developed for easy implementation of the proposed model. While analyzing our results, we found that through speciation and evolution, gene methylation (6mA) in regulatory sequences has changed (reduced frequency of occurrence). This could be an interesting and fundamental factor to be investigated thoroughly to answer the mechanism of gene birth as well as gene regulation. Moreover, the epigenetic control of growth and development along with stress tolerance and disease resistance could be addressed in the near future using the results obtained from our proposed model.

## Data availability statement

The original contributions presented in the study are included in the article/**Supplementary Material**. Further inquiries can be directed to the corresponding author.

## Author contributions

DS: Conceptualization, Data curation, Formal Analysis, Methodology, Software, Writing – original draft. TD: Data curation, Formal Analysis, Methodology, Software, Writing – original draft. KP: Data curation, Formal Analysis, Writing – original draft. MY: Formal Analysis, Software, Writing – original draft. SB: Conceptualization, Data curation, Formal Analysis, Writing – review & editing. SM: Conceptualization, Formal Analysis, Writing – original draft. DM: Conceptualization, Funding acquisition, Methodology, Writing – review & editing. SP: Software, Writing – review & editing. AR: Writing – review & editing. SA: Conceptualization, Methodology, Writing – original draft, Writing – review & editing.
