# Supplementary material for: MethSemble-6mA: an ensemble-based 6mA prediction server and its application on promoter region of LBD gene family in Poaceae
Source: Front Plant Sci. 2023 Oct 9;14:1256186. doi: 10.3389/fpls.2023.1256186 (PMC10591185; doi:10.3389/fpls.2023.1256186)
Supplement: Supplementary file 1 [file DataSheet_1.docx]

Supplementary Material

**1. Supplementary Data**

Uploaded separately with manuscript submission.

**2. Supplementary Figures and Tables**

**2.1 Supplementary Tables**

**S1: Performance of Three ML Models trained on Rice Dataset**

|  | **SVM** | **Random Forest** | **Gradient Boosting** |
| --- | --- | --- | --- |
| **Accuracy** | 91.47 | **92.06** | 90.76 |
| **Sensitivity** | 95.77 | **95.91** | 94.49 |
| **Specificity** | 83.98 | **85.22** | 83.97 |
| **MCC** | 81.44 | **82.64** | 79.63 |
| **AUC** | 91.59 | **92.09** | 90.41 |

**S2: Performance of Three ML Models trained on *Arabidopsis* Dataset**

|  | **SVM** | **Random Forest** | **Gradient Boosting** |
| --- | --- | --- | --- |
| **Accuracy** | 84.04 | **86.37** | 83.37 |
| **Sensitivity** | 96.31 | **96.56** | 96.28 |
| **Specificity** | 69.20 | **72.89** | 68.19 |
| **MCC** | 69.17 | **72.95** | 68.15 |
| **AUC** | 86.52 | **88.31** | 86.02 |

**S3:** **Performance of EpiSemble 2.0 with Existing Models**

|  | **MethSemble-6mA** | **EpiSemble** | **Meta-i6mA** |
| --- | --- | --- | --- |
| **Accuracy** | **93.12** | 90.94 | 92.09 |
| **Sensitivity** | 96.84 | 96.92 | **97.87** |
| **Specificity** | **86.55** | 81.44 | 82.9 |
| **MCC** | **85.01** | 80.91 | 83.42 |

**2.2 Supplementary Figures**

**(A)**


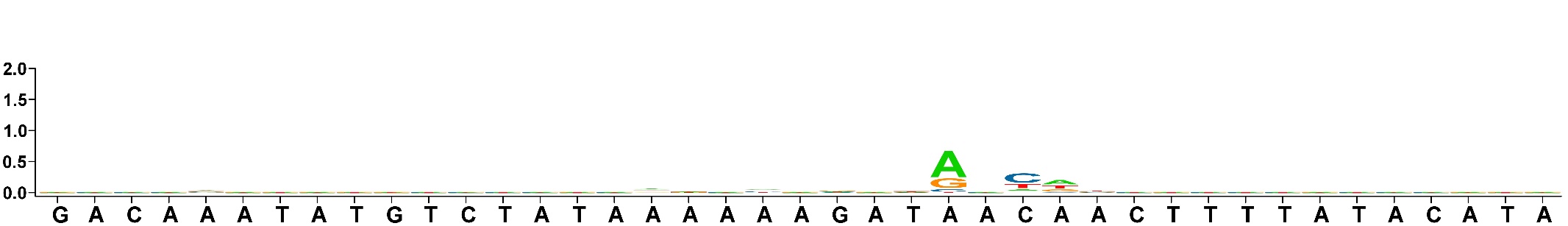


**(B)**


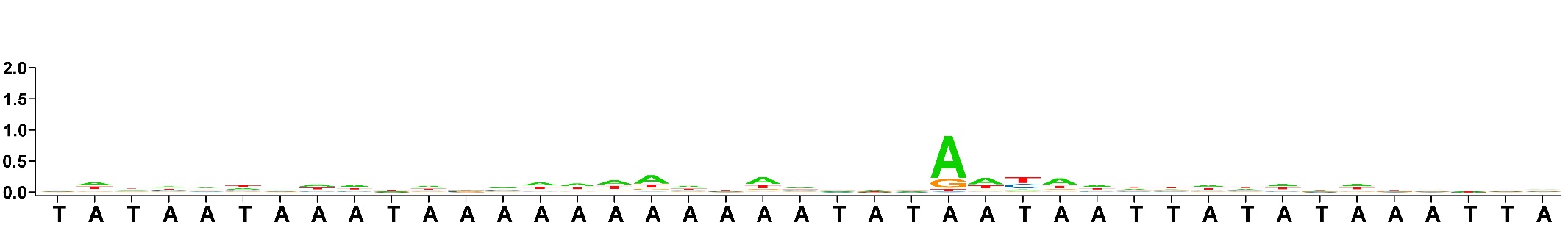


**(C)**


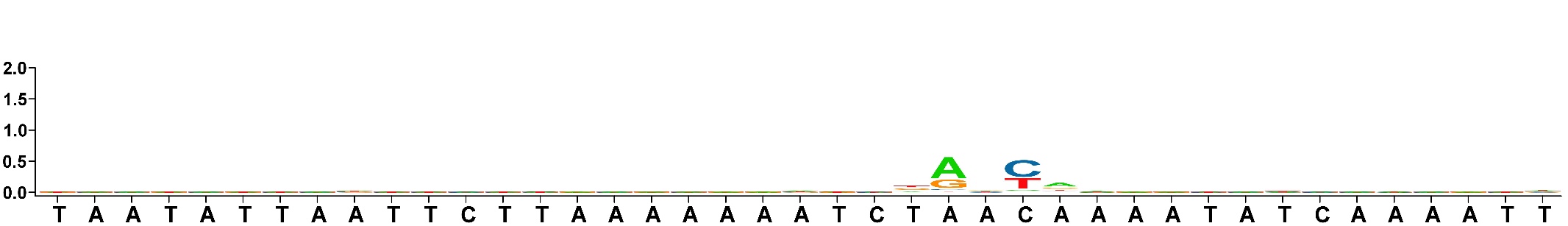


**(D)**


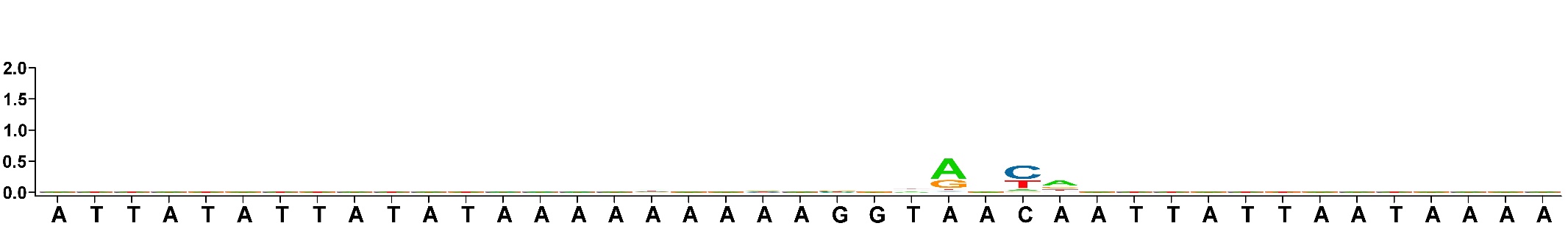


**(E)**


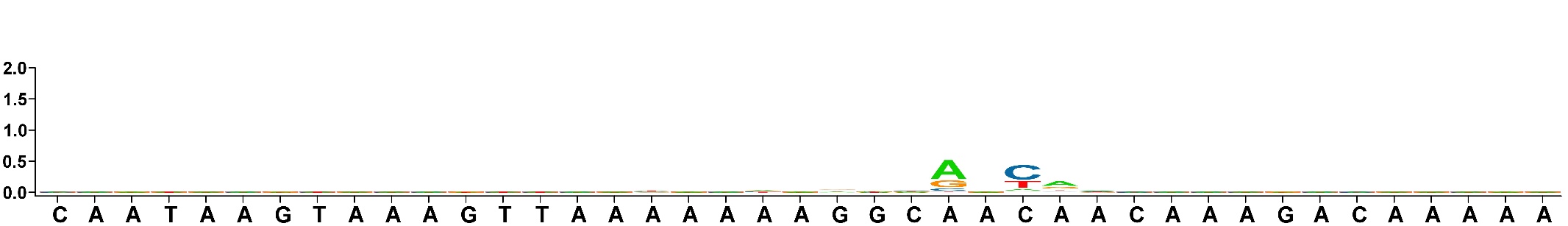


**(F)**


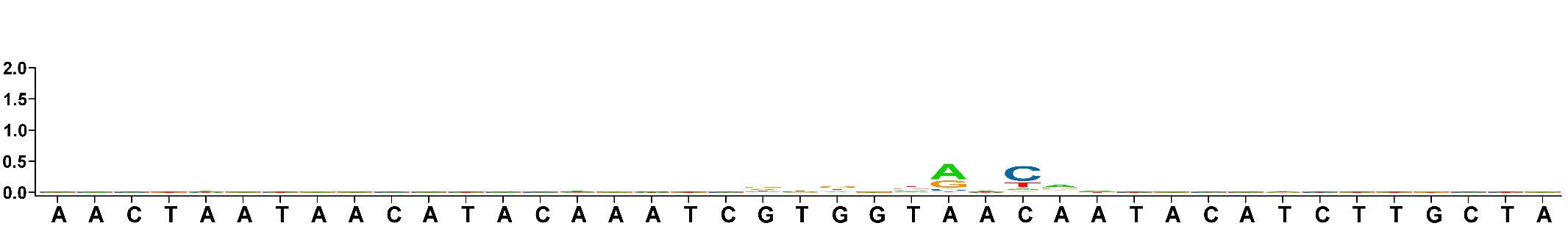


**Figure S1:** Enrichment analysis of 6mA sites in different species. A) *Aegilops tauschii,* B) *Arabidopsis thaliana,* C) *Oryza sativa,* D) *Triticum aestivum,* E) *Triticum dicoccoides,* F) *Triticum urartu*
